# A Mixture of Experts Model for Third-Party Pipeline Intrusion Detection Using DAS

**DOI:** 10.3390/s26061955

**Published:** 2026-03-20

**Authors:** Shenbin Zhu, Minglei Fu, Haifeng Zhang, Hongyuan Jiao, Yanhua Zhao, Zhengxiang Wu, Haiming Wang, Bohan Song

**Affiliations:** 1 College of Information Engineering, Zhejiang University of Technology, Hangzhou 310023, China; zhushenbin@viewshine.cn; 2 Zhejiang Viewshine Intelligent Meter Co., Ltd., Hangzhou 310023, China; zhaoyanhua@viewshine.cn (Y.Z.); wuzhengxiang@viewshine.cn (Z.W.); 3 School of Information and Control Engineering, Liaoning Petrochemical University, Fushun 113001, China; zhanghaifeng@lnpu.edu.cn (H.Z.); jiaohongyuan@126.com (H.J.); 15733823574@163.com (B.S.); 4 Intelligent Research Center, PipeChina Institute of Science and Technology, Tianjin 300450, China; wanghm01@pipechina.com.cn

**Keywords:** oil and gas pipeline, third-party intrusion, distributed acoustic sensing, Mixture of Experts, weak signal recognition

## Abstract

**Highlights:**

**What are the main findings?**

- Three specialized expert networks based on the Multi-Scale CNN (MS-CNN) architecture are proposed, each designed to recognize specific pipeline threat events (manual excavation, mechanical excavation, and heavy vehicle rolling). This approach addresses the limitations of single-model architectures in capturing diverse threat characteristics.
- A lightweight spatio-temporal perception gating network with weak signal detection is developed, serving as an intelligent scheduler that activates only the most appropriate expert model for each input signal, while incorporating a weak-signal detection branch that computes real-time SNR features and adjusts expert weights accordingly.

**What are the implications of the main findings?**

- The gating mechanism effectively reduces computational overhead while maintaining high recognition accuracy, making real-time deployment feasible even in resource-constrained environments.
- The integration of weak signal detection substantially enhances the accuracy of recognizing weak signals.

**Abstract:**

Distributed acoustic sensing (DAS) in pipeline safety warning systems confronts multiple challenges during technological evolution and application expansion, primarily including recognition accuracy, real-time performance, and the identification of weak signals for pipeline third-party intrusion (TPI) detection in complex environments. So, this paper proposes a Pipeline Fiber Optic Warning-Mixture of Experts (PFOW-MoE) method to address challenges in DAS systems. The proposed method is innovative in the sense that: (1) Multi-modal feature perception expert model design: Different intrusion behaviors are unique in the time, spatial, and frequency domains; (2) Efficient decision framework with dynamic gating mechanism: It evaluates input signal features in real time. (3) Robustness enhancement mechanism for weak signal perception: A weak signal detection branch is added to dynamic gating. Experimental validation on actual pipeline datasets shows PFOW-MoE achieves 98.27% accuracy on the entire sample set. On weak signal samples, it achieves 96.00%. The single-sample inference time is only 0.78 ms, meeting practical real-time engineering needs.

## 1. Introduction

Oil and gas transmission pipelines are core infrastructure in the energy supply chain. They directly impact national energy security and environmental protection. However, third-party intrusion (TPI) is now the main threat to pipeline safety [1]. As a result, there is an urgent need for high-precision, real-time, and adaptable pipeline security monitoring and warning technologies. Distributed acoustic sensing (DAS) systems have become the main technology for preventing third-party damage to oil and gas pipelines. Their advantages include long-distance continuous monitoring—covering more than 100 km with one device—meter-level spatial resolution, resistance to electromagnetic interference, and intrinsic safety [2,3]. DAS hardware technology is rapidly improving, enhancing signal acquisition and spatial coverage. However, the development of warning algorithms lags behind practical engineering needs. Oil and gas pipelines operate in complex environments, such as urban–rural junctions, mountainous areas, and water areas. Computing resources are often limited. Traditional algorithms struggle with recognition accuracy, real-time performance, and weak signal detection. There is an urgent need for intelligent warning algorithms with strong adaptability and high computational efficiency.

The evolution of research on pipeline fiber optic warning technology shows a significant trend. It has shifted from traditional signal processing to deep learning-driven intelligence. Early work mainly focused on methods such as Fourier analysis and wavelet transform [4,5]. These achieved event recognition through manually designed time-domain or frequency-domain features, such as energy, spectrum, and entropy. However, these methods rely heavily on expert experience, have limited anti-interference capabilities, and are difficult to adapt to complex, changing pipeline environments. With the rise in deep learning, algorithm research has gradually shifted toward automatic feature learning. Convolutional Neural Networks (CNNs) have become the core technology for pipeline fiber optic warning systems. This is due to their excellent ability to automatically extract features and capture local spatiotemporal correlations [6,7]. The multi-feature fusion CNN model proposed by Yang et al. achieved 95.86% recognition accuracy on real pipeline datasets. This result verifies the superiority of deep learning in feature learning [8]. Related research further focused on optimizing network architecture and multi-model fusion. The CNN+BiLSTM (Bidirectional Long Short-Term Memory) architecture combines the spatial feature extraction of CNNs with the temporal modeling capabilities of LSTM. This combination effectively solves the problem of modeling temporal dependencies in pipeline vibration signals. It is particularly suitable for capturing the temporal evolution of transient events [9,10]. To address the contradiction between computational efficiency and real-time performance, lightweight models such as Inception-DVS were developed, applying lightweight Inception networks to Distributed Vibration Sensing (DVS) recognition. These significantly reduce computational complexity while maintaining recognition accuracy by leveraging multi-branch convolutional structure designs [11,12]. To alleviate the issue of scarce labeled data in real scenarios, unsupervised feature learning methods like Autoencoders (AE) and Variational Autoencoders (VAE) have been introduced in pipeline warning research [13,14]. By pre-training on unlabeled data, the model can learn more generalized feature representations and improve adaptability to unseen pipeline environments.

Despite significant progress in pipeline fiber optic warning technology, three main challenges persist in long-term operation. First is the balance between model accuracy and generalization. Most existing methods use a single model architecture for all pipeline threat events. However, conflicts in multimodal features and mismatches in feature granularity make it hard for a single model to capture the diverse features of different intrusion types [15,16]. Second, there is a contradiction between computational overhead and real-time performance. To cover all event types, single models need to increase network depth and width. This prolongs inference time and struggles to meet the millisecond-level real-time requirements of fiber optic warning systems [17,18]. Third, robustness is insufficient in low signal-to-noise ratio (SNR) environments. Long-distance DAS system signals usually have low SNR. The surrounding environment of pipelines are also increasingly complex, with factors like traffic noise and industrial interference [19,20].

To address the above challenges, this paper proposes an optimized Mixture of Experts (MoE)-based fiber optic warning recognition method. This approach stands at the intersection of deep learning theory and practical engineering needs [21,22,23]. The goal is to achieve high-precision, real-time, and robust pipeline intrusion detection. The main innovations of this research are as follows:

(1) Multi-modal feature perception expert model design: Different intrusion behaviors, such as manual excavation, mechanical excavation, and heavy vehicle rolling, are unique in the time, spatial, and frequency domains. For these, multiple expert recognition models are designed using the multi-scale CNN (MS-CNN) model. By differentiating expert network structures, the system precisely recognizes various threat events. This approach effectively addresses the challenge of balancing accuracy and generalization with a single model.
(2) Efficient decision framework with dynamic gating mechanism: A lightweight spatio-temporal perception gating network acts as the model’s “intelligent scheduling center.” It evaluates input signal features in real time. Only the expert model best suited to the current signal characteristics is activated, reducing computational overhead. This preserves recognition accuracy while overcoming real-time deployment challenges for complex AI models in long-distance DAS systems.
(3) Robustness enhancement mechanism for weak signal perception: A weak signal detection branch is added to dynamic gating. It calculates the signal’s SNR features in real time and dynamically adjusts the weights output by optimal expert. This greatly reduces the missed-alarm rate for small vibration signals.

The rest of this paper is organized as follows. Section 2 describes the principles of fiber optic warning technology and the mixture of experts model. Section 3 explains the overall architecture of the MoE-based pipeline fiber optic warning method. Section 4 presents experimental verification of the method and field application. Section 5 summarizes research results and proposes future research directions.

## 2. Background and Related Work

### 2.1. Principle of Pipeline Fiber Optic Warning Technology

**Figure 1:**
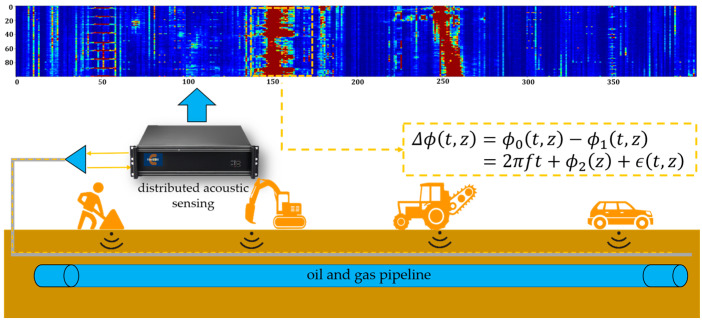
Structure of pipeline fiber optic warning based on distributed acoustic sensing.

PFOW technology typically uses redundant single-mode fiber in communication cables laid in the same trench as pipelines as the sensing medium, achieving spatial positioning and continuous time monitoring of vibration signals via phase-sensitive optical time-domain reflectometry (φ-OTDR) [24], as shown in Figure 1. When vibration events such as manual operation, mechanical excavation, and vehicle rolling occur around the pipeline, the disturbance can change fiber micro-bending loss, leading to modulation of the detection light phase. The phase change can be formally expressed as:

(1) $$\Delta \phi \left(t,z\right)=\phi_{0}\left(t,z\right)-\phi_{1}\left(t,z\right)=2\pi ft+\phi_{2}\left(z\right)+\epsilon \left(t,z\right)$$

where $\phi_{0}(t,z)$ and $\phi_{1}(t,z)$ are the phase values of the reference light and detection light at the space-time point (t,z) respectively; f is the characteristic frequency of the vibration source; $\phi_{2}(z)$ is the static phase offset term related to the spatial position; ϵ(t,z) represents random noise interference. Through demodulation, feature extraction, and intelligent recognition of this phase-difference signal, type discrimination and threat risk assessment around the pipeline can be achieved [25].

**Figure 2:**
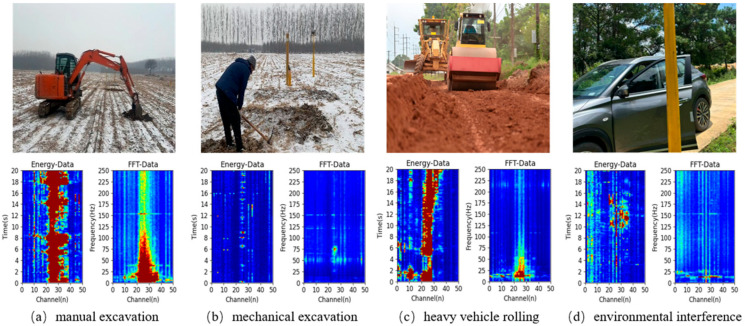
Four pipeline intrusion event categories: (**a**) manual excavation, (**b**) mechanical excavation, (**c**) heavy vehicle rolling, and (**d**) environmental interference. The lower side displays typical DAS signal characteristics for each event, using energy and frequency waterfall plots rendered as heatmaps. In all plots, the horizontal axis is the DAS channel number (n). The Energy-Data waterfall plot’s vertical axis shows time (s), while the FFT-Data plot’s vertical axis shows frequency (Hz). In the energy waterfall plots, red areas represent strong DAS time-domain energy signals, indicating intense vibration activities. In the frequency-domain waterfall plots, red regions denote the distribution of primary signal components, highlighting dominant frequency patterns. Notably, the DAS signals of heavy vehicle rolling events exhibit distinct characteristic peaks in the frequency domain of 25–50 Hz.

Based on the statistics of actual on-site monitoring data, typical vibration events along pipelines can be divided into four categories, as shown in Figure 2.

(1) Manual excavation intrusion: Involves personnel using manual tools (such as shovels, picks, etc.) to excavate above the pipeline, mostly for potential oil theft or deliberate sabotage. Such signals have the characteristics of low energy and intermittent impact, with lower signal amplitude and a narrower spatial influence range, usually with a radius < 10 m, making them the most easily missed event type in existing detection systems [26].
(2) Mechanical excavation intrusion: Mainly refers to illegal excavation activities of large construction equipment, such as excavators and drills, within the pipeline security protection zone. The DAS signal of this type of event shows characteristics of a high-amplitude impact and continuous vibration, with an energy-diffusion pattern centered on the operating point in space (usually with a radius > 50 m).
(3) Heavy vehicle rolling: Mainly describes the passage of trucks, construction machines, or harvesters over or near the pipeline. The signals include continuous, strong vibrations with frequency domain peaks at 25–50 Hz [27].
(4) Environmental Interference: Including non-threatening, harmless interference events such as normal traffic vehicles, rainfall and animal activities. Such signals are usually highly random and lack clear periodicity. Although they do not directly threaten pipeline safety, they significantly impact the detection efficiency and accuracy of AI models [28,29,30].

The main technical challenge in pipeline intrusion detection is efficiently distinguishing actual threat events (such as mechanical operations or manual excavation) from harmless interference in complex environmental noise, especially when the SNR is below 5 dB, while also satisfying the strict real-time requirements of practical engineering.

### 2.2. Principles of Mixture of Experts Model

Mixture of Experts (MoE) is an ensemble learning framework. It uses specialized submodels, called experts, and a scheduling mechanism (a gating network). Together, these components efficiently solve complex tasks [31,32]. The core idea is to break down complex problems into sub-tasks. A dedicated expert model handles each sub-task. The gating network then selects the most suitable expert for each input, improving computational efficiency while ensuring accuracy.

MoE architecture includes parallel expert networks and a gating network. Its mathematical representation is:

(2) $$\hat{y}\left(x\right)=\sum_{i=1}^{K}g_{i}(x)\cdot f_{i}\left(x\right)$$

where x is the input sample; K is the number of expert networks; $f_{i}(x)$ represents the output of the i expert network; $g_{i}(x)$ represents the weight assigned by the gating network to the i expert, satisfying the probability normalization constraint $\sum_{i=1}^{K}g_{i}(x)=1$ and $g_{i}(x)\geq 0$.

## 3. Research Method

**Figure 3:**
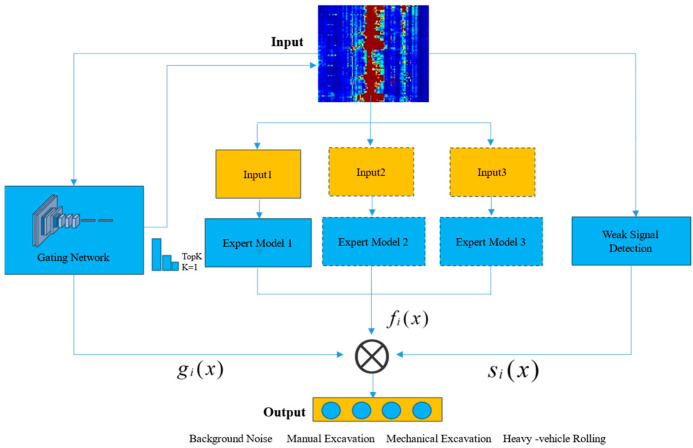
Structure of PFOW-MoE. First, DAS data passes through the dynamic gating mechanism to select the optimal expert model; second, specific dimensions of the DAS signal are extracted according to the chosen expert model requirements for model computation; third, weak signal weights are calculated; finally, the gating weights, expert model results, and weak signal weights are weighted and fused to output the final detection result.

This section addresses three core challenges identified in the introduction: balancing model accuracy and generalization, reconciling computational efficiency with real-time performance, and enhancing robustness in low signal-to-noise ratio environments. We propose a pipeline intrusion detection method based on a MoE model, named the Pipeline Fiber Optic Warning-Mixture of Experts (PFOW-MoE) method, as shown in Figure 3. This approach achieves precise identification of multiple types of intrusion events in complex pipeline environments. It does so through multi-expert model design, an efficient decision framework with a dynamic gating mechanism, and a robustness enhancement mechanism for weak signal perception [33].

### 3.1. Mixture of Experts Model Design

**Figure 4:**
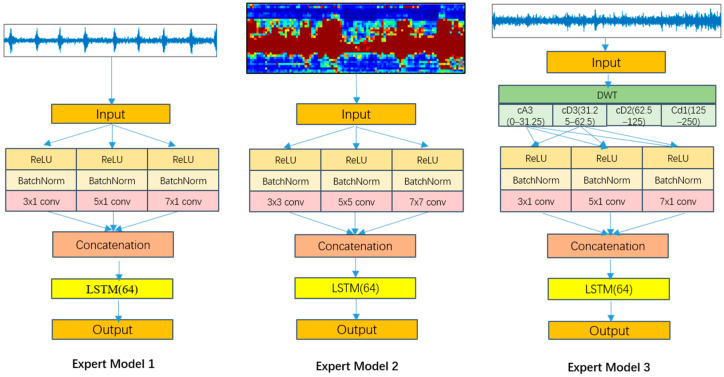
Structure of expert models. Expert model 1 for manual excavation event recognition, expert model 2 for mechanical excavation event recognition, and expert model 3 for heavy vehicle rolling event recognition.

This section designs three expert models for pipeline intrusion detection: Expert model 1 for manual excavation event recognition, expert model 2 for mechanical excavation event recognition, and expert model 3 for heavy vehicle rolling event recognition. Their specific architectures are illustrated in Figure 4.

#### 3.1.1. Expert Model 1: Manual Excavation Recognition

Expert model 1 is designed for identifying low-energy, intermittent impact features of manual excavation events, employing a hybrid architecture combining multi-scale 1D Convolutional Neural Network (MS-1dCNN) with Long Short-Term Memory (LSTM) network. For DAS signals from a specific spatial channel $z_{i}$, denoted as $x(t,z_{i})=\phi (t,z){|}_{z=z_{i}}$, the MS-1dCNN extracts multi-scale features in parallel using three convolution kernels (3 × 1, 7 × 1, 11 × 1), corresponding to impact peaks (small scale), energy decay (medium scale), and periodic variations (large scale).

The multi-scale convolution operation is given by:

(3) $$H_{\left(l\right)}=\mathrm{Concat}{\left(\mathrm{ReLU}\left(\mathrm{BN}\left(W_{k}^{\left(l\right)}\times X^{\left(l-1\right)}+b_{k}^{\left(l\right)}\right)\right)\right)}_{k\in \left\{3\times 1,7\times 1,11\times 1\right\}}$$

Subsequently, an LSTM layer models the temporal evolution:

(4) $$L_{t}=\mathrm{LSTM}\left(H_{t},L_{t-1}\right)$$

This model effectively integrates multi-scale features with long-range temporal dependencies, making it particularly suitable for recognizing intermittent impact signals, such as shovel excavation, with a binary classification indicating whether it is manual excavation [34,35].

#### 3.1.2. Expert Model 2: Mechanical Excavation Recognition

Expert model 2 targets the spatial-temporal energy diffusion characteristics of mechanical excavation events, adopting a multi-scale 2D Convolutional Neural Network (MS-2dCNN) combined with LSTM architecture. For multi-channel spatial-temporal signals $X\in R_{T\times Z}$ acquired by the DAS system (where T is the number of time steps and Z is the number of spatial channels), the model simultaneously extracts multi-scale features in both spatial and temporal dimensions using 3 × 3, 5 × 7, and 7 × 7 convolution kernels:

(5) $$H^{\left(l\right)}=\mathrm{Concat}{\left(\mathrm{ReLU}\left(\mathrm{BN}\left(W_{k}^{\left(l\right)}\times X^{\left(l-1\right)}+b_{k}^{\left(l\right)}\right)\right)\right)}_{k\in \left\{3\times 3,5\times 5,7\times 7\right\}}$$

The convolution output is followed by an LSTM layer to capture long-term dependencies:

(6) $$\;\;L_{t}=\mathrm{LSTM}\;\left(H_{t}^{\left(L\right)},L_{t-1}\right)$$

This model provides robust feature extraction capabilities for high-energy, widely diffused mechanical excavation events, producing binary classification results.

#### 3.1.3. Expert Model 3: Heavy-Vehicle Rolling Recognition

Expert model 3 adopts a hybrid architecture of “discrete wavelet transform (DWT), multi-scale 1D convolution (MS-1dCNN) and LSTM” to address the low-frequency periodic vibration characteristics of heavy vehicle rolling events. For a one-dimensional time series signal x(t,z) from a specified spatial channel z, the model first decomposes the original signal through a three-level discrete wavelet transform:

(7) $$X_{\left(l,i\right)}\left(t,z\right)={DWT}_{\left(l,i\right)\;}\left\{x\left(t,z\right)\right\}$$

where l denotes the decomposition level, and i represents the frequency band index within each level.

Considering that the main energy of heavy vehicle rolling events is concentrated in the low-frequency band, multi-scale one-dimensional convolution is used to extract features from different receptive fields in parallel for the first and second frequency band signals (25–50 Hz) in the third-level decomposition:

(8) $$F_{\left(3,i\right)}\;\left(t,z\right)=\mathrm{Concat}{\left(\mathrm{ReLU}\left(\mathrm{BN}\left(W_{k}^{\left(3,i\right)}\times X^{\left(3,i\right)}\left(t,z\right)+b_{k}^{\left(3,i\right)}\right)\right)\right)}_{k\in \left\{3\times 1,5\times 1,7\times 1\right\}},\;\;\;i=1,2$$

Subsequently, the features from the first and second frequency bands after third-layer decomposition are concatenated and then input to an LSTM to model temporal dependencies:

(9) $$L_{t}=\mathrm{LSTM}\left(\mathrm{Concat}\left\{F_{\left(3,2\right)}\left(t,z\right),F_{\left(3,3\right)}\left(t,z\right)\right\},{\;L}_{t-1}\right)$$

Binary classification results are generated through feature fusion:

(10) $$y\;\left(t,z\right)=\mathrm{Softmax}\left(W_{o}\cdot L_{t}+b_{o}\right)$$

By combining frequency domain decomposition with temporal modeling, this model effectively captures the frequency patterns of heavy vehicle engine vibration and the periodic ground vibration characteristics [36,37], with a binary classification output.

### 3.2. Dynamic Gating Mechanism with Weak Signal Perception

To realize intelligent scheduling of expert models and solve the recognition problem in low SNR environments, this section proposes a dynamic gating mechanism with weak signal perception. As the core component of the PFOW-MoE framework, this mechanism achieves enhanced recognition of weak signals through the following two key steps:

#### 3.2.1. Lightweight Spatio-Temporal Perceptual Gating Network Design

To achieve precise scheduling of multi-expert models and efficient utilization of computing resources, this section designs a lightweight gating network architecture for the spatio-temporal characteristics of DAS signals. Unlike the common fully connected layer-based gating design in existing MoE architectures, the lightweight gating network directly takes the original 2D distributed acoustic sensing signal $X\in R_{T\times Z}$ (where T is the time series length and Z is the number of spatial channels) as input, and learns discriminative feature representations directly from raw spatio-temporal data through a dedicated Convolutional Neural Network structure to achieve adaptive matching between input signals and expert models [38,39].

The model extracts spatio-temporal feature representations simultaneously in the time dimension and spatial dimension by deploying a 7 × 3 size convolution kernel. For 2D DAS signal input $X\in R_{T\times Z}$, the convolution operation can be formally described as:

(11) $$H_{\left(l\right)}=\mathrm{ReLU}\left(\mathrm{BN}\left(W_{k}^{\left(l\right)}\times X^{\left(l-1\right)}+b_{k}^{\left(l\right)}\right)\right)$$

First, it extracts spatio-temporal features from the original DAS signal through stacked spatio-temporal convolution layers, then compresses multi-dimensional features into a fixed-dimensional feature vector $f_{gate}$ through Global Average Pooling (GAP), and finally converts the feature vector into expert weight allocation probabilities through fully connected layers and Softmax functions:

(12) $$g=\mathrm{Softmax}\left(W_{o}\cdot f_{gate}+b_{o}\right)$$

where $g=[g_{1},g_{2},g_{3}]$ represents the weight vector assigned to the three expert models, satisfying the probability normalization constraint $\sum_{i=1}^{3}g_{i}=1$.

This section adopts a Winner-Takes-All strategy by selecting the single expert with the highest weight among K = 3 expert models, that is, using a K = 1 decision mode. After normalizing the expert weight vector g from the gating network, the expert i with the maximum weight is chosen, and only this expert is forward-propagated. The resulting classification output y is then calculated as follows:

(13) $$y=g_{i}\left(x\right)\cdot f_{i}\left(x\right)$$

Here, $g_{i}(x)$ is the gating weight of the chosen expert i, and $f_{i}(x)$ is this expert’s prediction for input x.

To validate the effectiveness and rationality of the Winner-Takes-All strategy in the gating mechanism, this section designed comparative experiments for the PFOW-MoE model. The experiments explored different K value configurations within the TOP-K gating strategy and included comprehensive performance evaluations from multiple dimensions. Detailed experimental results will be presented in the Section 4.

Simultaneously, to enhance the PFOW-MoE model’s capability in addressing unknown events, the proposed gating network is designed to automatically label an event as “unknown” and preserve the original DAS data when the maximum value of the output expert weight vector (g) falls below a 0.5 threshold. This mechanism provides essential support for subsequent analysis and model extension. For the incorporation of newly emerging event types, it is sufficient to design and train new expert models, integrate them into the MoE framework, and fine-tune the output layer of the gating network to $g=[g_{1},g_{2},g_{3},g_{4}]$ to achieve adaptation to the new experts. This incremental expansion strategy not only maintains system stability but also markedly enhances the integration efficiency of new event types. Within this gating network architecture, even as the number of experts increases, only the output layer parameters of the gating network require adjustment. Consequently, the computational complexity remains independent of the total number of experts, thereby ensuring the stability of the model and efficient real-time computational performance.

In particular, the 7 × 3 convolution kernel design in gating network has clear physical significance and technical innovation. The 7 sampling points in the time dimension correspond to a time window of approximately 1.5 s in the DAS system. This window length is carefully set to capture the short-term dynamic features of pipeline intrusion events, such as the impact initiation stage of mechanical excavation or the single tapping action of manual excavation. At the same time, it avoids redundant information that could occur with longer time windows. The 3-channel design in the spatial dimension can adaptively capture the local correlation of adjacent spatial positions, reflecting the spatial diffusion characteristics of pipeline vibration signals. This design fully utilizes the spatio-temporal coupling characteristics of DAS signals.

#### 3.2.2. Recognition Enhanced by Weak Signal Detection

To address the challenge of degraded model perception performance in low signal-to-noise ratio (SNR) environments, this section proposes a lightweight weak-signal detection enhancement strategy that directly participates in adjusting the expert model’s output weight via integrated physical-layer SNR calculation. This strategy calculates the signal power and noise power for each channel z, and then computes the real-time SNR for each channel. The calculation formulas are as follows:

The signal power for channel z is calculated as:

(14) $$P_{signal}(z)=\sum_{t=1}^{T}|x(t,z){|}^{2}$$

The noise power for channel z is calculated as:

(15) $$P_{noise}(z)=\sum_{t=1}^{T}|x_{noise}(t,z){|}^{2}$$

The SNR for channel z is then computed as:

(16) $$SNR(z)=10\log_{10}\left(\frac{P_{signal}(z)}{P_{noise}(z)}\right)$$

where T is the total number of time samples, x(t,z) is the current measured signal at time t and channel z, and $x_{noise}\left(t,z\right)$ is the background noise reference signal at time t and channel z.

Based on the real-time computed signal-to-noise ratio (SNR) for each channel z, the gating mechanism dynamically optimizes the probability of the final classification output y through a nonlinear transformation. During this optimization process, an SNR-aware gain factor $s\left(z\right)$ is introduced for each channel, which is defined as follows:

(17) s(z)=1+α⋅Sigmoid(β×(γ−SNR(z)))

The calculation formula for the final classification output $\hat{y}\left(z\right)$ for channel z is given by:

(18) $$\hat{y}\left(z\right)=y\left(z\right)\cdot s\left(z\right)$$

Here, $y\left(z\right)$ represents the original classification output for channel z, and $s\left(z\right)$ denotes the dynamic gain factor based on SNR for channel z. The hyperparameters are set as follows: α is the output adjustment strength hyperparameter, β is the SNR sensitivity adjustment parameter, and γ is the weak signal threshold.

The core mechanism of this dynamic adjustment strategy is its ability to map SNR differences for each channel z to nonlinear gain factors. It uses the Sigmoid function when the input signal’s SNR for a channel approaches or falls below the weak signal threshold γ. This approach effectively amplifies weight differentiation between experts for each channel. As a result, it enhances the expert model output weights under low SNR conditions for individual channels. This design achieves direct fusion between physical layer signal quality metrics and the deep learning model’s decision process. Through a parameterized nonlinear transformation, it targets perception enhancement for weak signals across all channels. At the same time, it maintains the gating mechanism’s computational lightweight character.

### 3.3. Model Inference Process

The inference process of the PFOW-MoE model can be summarized as: first, construct expert models for different intrusion event types (including manual excavation, mechanical excavation, and heavy vehicle rolling event recognition models); the weak signal detection branch calculates SNR based on the direct power ratio method of input signals and background noise, and maps it to weak signal detection probability $p_{weak}$; then, the lightweight spatio-temporal perception gating network directly extracts spatio-temporal features from the raw DAS signals to compute initial gating weights g and selects the optimal expert model according to the Winner-Takes-All strategy. This expert model outputs threat recognition event category predictions y by calculating the DAS signal features it specializes in. Meanwhile, the weak signal detection mechanism obtains the SNR perception gain factor for the current signal by calculating the SNR. Finally, the gating weights g, event category predictions y, and gain factor s are multiplied to obtain the final prediction result $\hat{y}$. The specific algorithm flow is shown in Algorithm 1.
**Algorithm 1. PFOW-MoE Inference Framework****Input:** DAS signal $x(t,Z)=\{\phi (t,z_{1}),\phi (t,z_{2}),\ldots ,\phi (t,z_{n})\}$, background noise reference signal $x_{noise}(t,Z)=\{\phi (t,z_{1}),\phi (t,z_{2}),\ldots ,\phi (t,z_{n})\}$ **Output:** Classification result $\hat{y}$ 1: Expert model construction 2: Expert_Model_1: Manual excavation event recognition (MS-1dCNN + LSTM) 3: Expert_Model_2: Mechanical excavation event recognition (MS-2dCNN + LSTM) 4: Expert_Model_3: Heavy vehicle rolling event recognition (DWT + MS-1dCNN + LSTM) 5: Dynamic gating calculation 6: h=Conv2D(X,kernel_size=(7,3)) 7: $f_{gate}=\mathrm{GlobalAvgPool}(h)$ 8: $g=\mathrm{Softmax}(W_{o}\cdot f_{gate}+b_{o})$ 9: Expert selection 10: $i^{*}=\arg\max(g_{1},g_{2},g_{3})$ 11: Execute expert model for classification 12: For each spatial channel $z=z_{1}$ to $z_{n}$: 13: $\;\;\;\;\mathrm{if}\;i^{*}(z)==1$: 14: $\;\;\;\;\;\;\;\;y(z)=g_{1}\cdot Expert\text{\_}Model\text{\_}1(x(t,z))$ 15: $\;\;\;\;\mathrm{elif}\;i^{*}(z)==2$: 16: $\;\;\;\;\;\;\;\;y(z)=g_{2}\cdot Expert\text{\_}Model\text{\_}2(x(t,z))$ 17:     else: 18: $\;\;\;\;\;\;\;\;y(z)=g_{3}\cdot Expert\text{\_}Model\text{\_}3(x(t,z))$ 19: Weak signal detection 20: For each spatial channel $z=z_{1}$ to $z=z_{n}$: 21: $\;\;\;\;P_{signal}(z)=\sum_{t=1}^{T}|x(t,z){|}^{2}$ 22: $\;\;\;\;P_{noise}(z)=\sum_{t=1}^{T}|x_{noise}(t,z){|}^{2}$ 23: $\;\;\;\;SNR(z)=10\log_{10}\left(\frac{P_{signal}(z)}{P_{noise}(z)}\right)$ 24:   s(z)=1+α⋅Sigmoid(β×(γ−SNR(z))) 25: Adjust identification results 26: For each spatial channel $z=z_{1}$ to $z_{n}$: 27: $\;\;\;\hat{y}(z)=y(z)\cdot s(z)$ 28: Return $\hat{y}$


## 4. Experiments and Results

### 4.1. Experimental Setup

**Figure 5:**
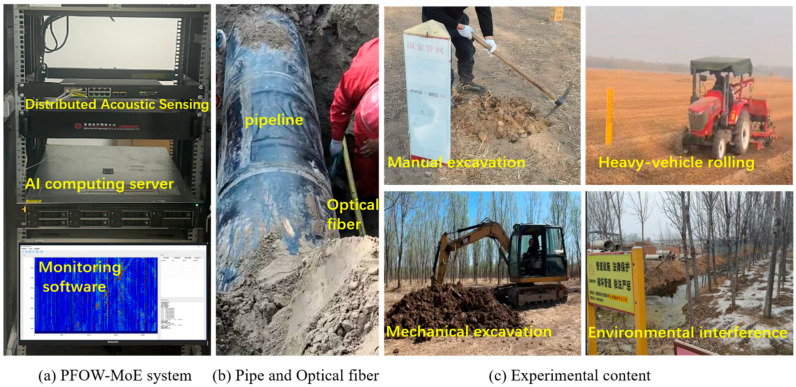
Experimental setup configuration.

The experiments were conducted on an in-service long-distance oil pipeline in the Shandong section of PipeChina. A bidirectional detection DAS system was installed at an intermediate station, enabling continuous monitoring of about 110 km. The pipeline travels through complex environments. It crosses multiple urban areas, rivers, and railway lines, and encounters superposition of multiple noise sources and low signal-to-noise ratio conditions: details are shown in Figure 5.

A total of 10 TB of original fiber-phase data were collected during the experiment. After signal preprocessing, segment labeling, and data augmentation, 5000 sets of labeled sample data were generated. This includes 1000 sets of weak-signal sample data (SNR < 5 dB). The samples cover four categories: interference noise, manual excavation, mechanical operation, and heavy vehicle rolling. The specific data distribution is shown in Table 1. All data were split into training, validation, and test sets at 70%:15%:15%.

The model training and evaluation were carried out on a computing platform equipped with a NVIDIA GeForce RTX 3090 GPU (NVIDIA Corporation, Santa Clara, CA, USA), an Intel(R) Xeon(R) Platinum 8260C CPU (2.30 GHz, Intel Corporation, Santa Clara, CA, USA), and 32 GB DDR4 memory, with the Linux operating system (Ubuntu 24.04) and Python 3.10 and TensorFlow 2.11.0 as the software development environment.

We comprehensively evaluated the performance of the PFOW-MoE model in pipeline intrusion detection tasks using five evaluation indicators:

1. Accuracy (Acc): Measures the overall classification correctness of the model and is calculated as:

Accuracy = (TP + TN)/(TP + TN + FP + FN)

Here, TP (true positive) represents the number of correctly identified threat events, TN (true negative) represents the number of correctly identified non-threat events, FP (false positive) represents the number of non-threat events falsely reported as threats, and FN (false negative) represents the number of threat events missed by the detection system.

2. False alarm rate (FAR): Measures the proportion of non-threat events that are incorrectly classified as threat events by the model.

FAR = FP/(FP + TN)

3. False negative rate (FNR): Measures the proportion of threat events that are incorrectly classified as non-threat events by the model.

FNR = FN/(TP + FN)(19)

4. F1-Score: The harmonic mean of precision and recall, which comprehensively evaluates the classification performance of the model.

F1-Score = 2 × (Precision × Recall)/(Precision + Recall)(20)

5. IT: Time for a single inference, in milliseconds.

### 4.2. Experimental Results and Analysis

Table 2 compares the performance of the PFOW-MoE model with current advanced pipeline intrusion detection methods across the entire sample set and the weak signal sample set.

All models are assessed using the same indicators. This guarantees fairness and comparability of the results. The experiment’s dataset includes various intrusion event samples from real pipeline environments. These diverse samples completely reflect the models’ performance in practical applications.

Table 2 clearly shows that the PFOW-MoE model significantly surpasses existing advanced methods across all metrics. Specifically, for overall sample performance, PFOW-MoE (with weak signal detection) achieves an accuracy of 98.27%, which is 6.54 percentage points higher than the MS-CNN model. The F1 score is 0.98, 0.06 points higher than the MS-CNN model. The false alarm rate is only 2.37%, and the false negative rate drops to 1.21%, effectively solving the practical issues mentioned in the introduction.

In terms of weak signal detection performance, on the low SNR sample set, PFOW-MoE (with weak signal detection) achieves an accuracy of 96.00%, which is 5.33 percentage points higher than the best comparison model, CNN+BiLSTM. Its F1 score is 0.97, 0.09 higher than the CNN+LightGBM model. The false alarm rate is 3.33%, and the false negative rate is 4.44%. These results are significantly better than those of other models, fully verifying the effectiveness of the weak signal detection branch.

Ablation experiment analysis further emphasizes the importance of the weak signal detection branch. Comparing PFOW-MoE with and without weak signal detection shows that the overall sample set accuracy improves by 0.80 points, while the accuracy for weak signal samples increases by 3.33 points. F1 scores also rise by 0.01 for the total sample set and 0.03 for the weak signal sample set, respectively, highlighting the critical role of the weak-signal detection branch in enhancing model performance. Additionally, the inference time of PFOW-MoE is only 0.78 ms, just 0.09 ms more than that of the CNN+BiLSTM model. This indicates that the gating network effectively balances the trade-off between accuracy and computational cost, meeting the real-time requirements of pipeline monitoring systems.

**Figure 6:**
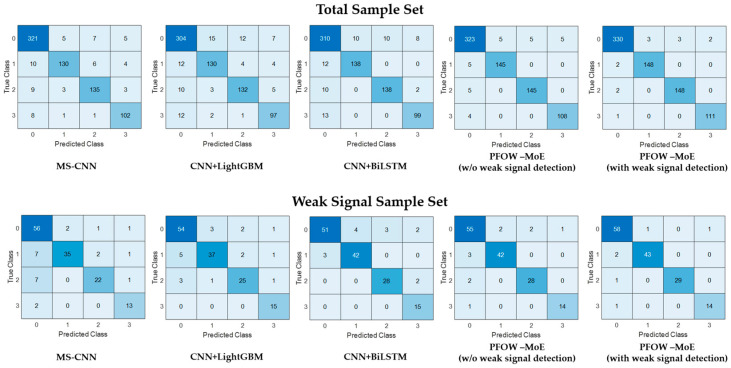
Confusion matrices of prediction results for multiple models. The confusion matrices show the classification performance of five recognition models on both the total sample set and the weak-signal sample set. In each matrix, the rows represent the true class and the columns represent the predicted class. The numbers 0–3 correspond to four event types: interference noise, manual excavation, mechanical operation, and heavy vehicle rolling.

Figure 6 presents the confusion matrices for each model, depicting their performance in recognizing the four event categories under both the total sample set and the weak signal sample test set. This provides a comprehensive visualization of classification effectiveness across varying signal conditions.

Regarding interference noise recognition (Event Category 0), the PFOW-MoE model exhibits marked advantages. Among the 338 interference noise samples in the total sample set, the PFOW-MoE model with weak signal detection correctly identifies 330 samples. The version without weak signal detection achieves 323 correct identifications, whereas the best-performing traditional model (MS-CNN) identifies only 321 samples correctly.

Among the 60 interference-noise samples in the weak-signal sample set, the PFOW-MoE model with weak-signal detection correctly identifies 58 samples and records only 2 false alarms. The version without weak signal detection correctly identifies 55 samples, resulting in 5 false alarms. In contrast, traditional models generally produce more false alarms. These findings suggest that the PFOW-MoE model, particularly the version incorporating weak signal detection, is highly effective in filtering non-threatening interference noise. This capacity substantially reduces the frequency of false alarms and enhances the model’s overall reliability. For the three threat events—manual excavation (Event Category 1), mechanical operation (Event Category 2), and heavy vehicle rolling (Event Category 3)—the PFOW-MoE model with weak signal detection demonstrates near-perfect classification performance. In comparison, traditional models fall short of achieving results as good as those achieved by the model.

These outcomes provide further validation that the expert model-based design framework enables more accurate identification of distinct threat event categories. This approach effectively leverages the domain-specific strengths of each expert submodel, thereby improving overall system performance.

### 4.3. Hyperparameter Optimization and Analysis

#### 4.3.1. Performance Comparison of Gating Strategies

To evaluate the performance of various gating strategies under weak signal conditions, a comparative experiment was conducted. This experiment assessed the effectiveness of weak signal enhancement methods and multi-expert fusion strategies TOP-K (K = 1, 2, 3) using data with a low signal-to-noise ratio (SNR < 5 dB). A total of 150 groups of weak signal samples were tested. Each K-value strategy was assessed both with and without the signal detection enhancement mechanism. The evaluation metrics included accuracy (classification correctness) and processing time (real-time performance). Table 3 presents the performance results of different K-value gating strategies on weak signal sample set.

This study conducted a comparative analysis of expert gating strategies with different TOP-K values in low signal-to-noise ratio environments, and evaluated the improvement achieved by incorporating the weak signal enhancement mechanism. The results indicate that the weak signal enhancement mechanism consistently yields superior identification performance compared to configurations without enhancement, as measured by accuracy, false alarm rate, false negative rate, and F1 score. These findings confirm that the weak signal enhancement mechanism effectively improves model identification capability under challenging SNR conditions. Furthermore, when the enhancement mechanism is applied, the performance metrics for different K values (1, 2, 3) become identical, suggesting that the combination of the enhancement mechanism and gating network fully utilizes signal features, allowing optimal identification even with K = 1. However, computational analysis reveals a substantial increase in processing time from 0.78 ms to 2.31 ms as the K value increases. Therefore, considering both performance and computational efficiency, the “weak signal enhancement mechanism + K = 1” (Winner-Takes-All) strategy is recommended. This approach achieves 96% accuracy while maintaining the lowest computation time (0.78 ms), providing an optimal balance between performance and efficiency.

#### 4.3.2. Analysis of Weak Signal Enhancement Hyperparameters

To systematically assess the impact of the weak signal enhancement mechanism on model performance, we conducted experiments comparing model outcomes across various α and β parameter combinations. The parameter α varied from 0.1 to 1.0 in increments of 0.1, while β ranged from 0.2 to 2.0 in increments of 0.2, yielding a total of 100 parameter combinations.

**Figure 7:**
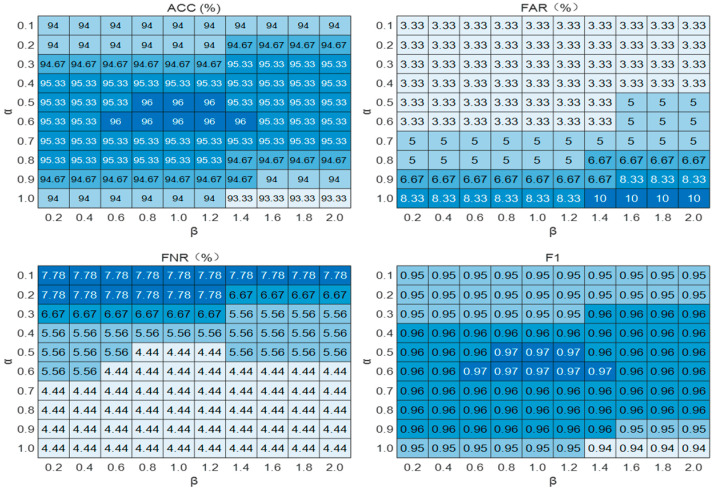
Heatmaps of model performance metrics for different α and β combinations in weak signal enhancement.

The test dataset comprised 15% of the weak signal sample set, totaling 150 data groups. Model detection capability was comprehensively evaluated using accuracy (ACC), false alarm rate (FAR), false negative rate (FNR), and F1 score, based on the PFOW-MoE configuration (incorporating weak signal detection, TOP-K = 1). A grid search was employed to identify the optimal α and β combination that maximized system detection performance. The corresponding evaluation metrics are presented in Figure 7.

Experimental results demonstrate that the parameter α exerts a distinct nonlinear influence on model performance. As α increases from 0.1 to 0.6, both accuracy and F1 score gradually improve, while the false negative rate (FNR) declines. However, when α exceeds 0.6, accuracy and F1 score decrease, and the false alarm rate (FAR) rises markedly. These findings suggest that excessively large α values render the system overly sensitive to noise, resulting in an increased incidence of false alarms.

A similar trend is observed for parameter β. As β increases from 0.2 to 1.0, accuracy and F1 score consistently improve. When β exceeds 1.0, both accuracy and F1 score decline, while FAR gradually increases. This outcome indicates that excessively large β values over-amplify noise signals, thereby diminishing system stability.

Grid search analysis identified the optimal parameter combination for the weak signal enhancement mechanism as α = 0.6 and β = 0.8–1.0. Under this configuration, the system achieves optimal performance, with a maximum accuracy of 96.00%, a minimum FAR of 3.33%, a minimum FNR of 4.44%, and a maximum F1 score of 0.97. This parameter combination is well suited to weak signal detection in pipeline intrusion scenarios, enabling effective discrimination between interference noise and genuine threat events, and providing a robust reference for practical implementation.

### 4.4. Field Application Effect

**Figure 8:**
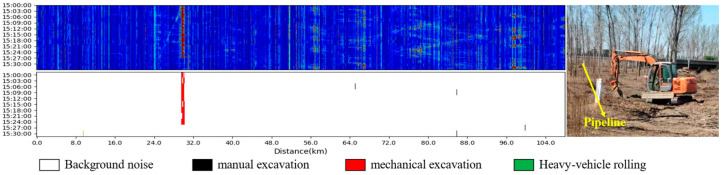
Application effect demonstration. The figure shows the PFOW system’s monitoring performance from 15:00 to 15:30 on 28 June 2025. In the identification diagram, mechanical excavation events appear in red. The detected event lasted about 30 min. On-site verification confirmed that the system detected an excavator performing unauthorized construction near the pipeline. Relevant personnel arrived quickly to intervene. Their actions prevented a potential pipeline security threat.

The PFOW-MoE model was deployed to a 110 km oil pipeline in China National Petroleum Pipeline Network Group (Beijing, China) for actual application testing, as shown in Figure 8. During 3 months of continuous operation, the system detected and warned of multiple real third-party intrusion events. Figure 8 shows the monitoring effect in actual deployment, including a feature waterfall plot of the entire line and a recognition result waterfall plot, which intuitively presents the model’s accurate recognition of intrusion events.

## 5. Conclusions

In this paper, we propose a novel PFOW-MoE method to effectively address third-party intrusion detection in complex pipeline environments. First, through task-specific expert model design, specialized recognition networks were built to capture the signal characteristics of different types of intrusion events, achieving precise classification of threat events such as manual excavation, mechanical excavation, and heavy-vehicle rolling. Then, the decision framework, designed based on the weak signal perception dynamic gating mechanism, by fusing multidimensional features and adaptively adjusting expert output weights, significantly improved warning accuracy in low-SNR environments while addressing the key bottleneck of computational overhead that restricts system real-time performance. Finally, comparative experiments with advanced methods on real pipeline datasets achieved superior detection performance (overall sample accuracy of 98.27%, 6.54 percentage points higher than the best single-model CNN; weak signal sample accuracy of 96.00%, 5.33 percentage points higher than the best comparison model CNN+BiLSTM). The proposed PFOW-MoE model can greatly improve the accuracy and real-time performance of pipeline security warning systems, effectively reduce the burden of manual monitoring, and thus promote the intelligent, digital operation of oil and gas pipeline monitoring systems.

In the future, we will focus on obtaining real data in areas such as pipeline leakage, pipeline internal detector positioning, and perimeter security. We will add new expert models to achieve broader threat event monitoring. These steps will further expand the application prospects of this method in energy infrastructure security protection.

## Figures and Tables

**Table 1:** Dataset distribution statistics.

Sample Type	Interference Noise	Manual Excavation	Mechanical Operation	Heavy Vehicle Rolling	Total
Total Sample Set Size	2250	1000	1000	750	5000
Weak Signal Sample Set Size	400	300	200	100	1000

**Table 2:** Performance comparison of different models in pipeline intrusion detection tasks.

Models	ACC (%)	FAR (%)	FNR (%)	F1-Score	IT (ms)
Total Sample Set					
MS-CNN [11]	91.73	5.03	6.68	0.93	0.54
CNN+LightGBM [8]	88.40	10.06	8.44	0.90	0.62
CNN+BiLSTM [9]	91.33	8.28	8.75	0.92	0.70
PFOW-MoE(w/o weak signal detection)	97.47	4.44	3.32	0.97	0.72
PFOW-MoE(with weak signal detection)	98.27	2.37	1.21	0.98	0.78
Weak Signal Sample Set					
MS-CNN [11]	84.00	6.67	17.78	0.85	0.54
CNN+LightGBM [8]	87.33	10.00	9.41	0.88	0.61
CNN+BiLSTM [9]	90.67	15.00	5.56	0.92	0.69
PFOW-MoE(w/o weak signal detection)	92.67	8.33	6.67	0.94	0.71
PFOW-MoE(with weak signal detection)	96.00	3.33	4.44	0.97	0.78

**Table 3:** Performance comparison of gating strategies on weak signal sample set.

Models	ACC (%)	FAR (%)	FNR (%)	F1-Score	IT (ms)
PFOW-MoE(w/o weak signal detection) (top-k = 1)	92.67	8.33	6.67	0.94	0.74
PFOW-MoE(w/o weak signal detection) (top-k = 2)	93.33	8.33	5.56	0.94	1.43
PFOW-MoE(w/o weak signal detection) (top-k = 3)	94.00	8.2	4.49	0.95	2.21
PFOW-MoE(with weak signal detection) (top-k = 1)	96.00	3.33	4.44	0.97	0.78
PFOW-MoE(with weak signal detection) (top-k = 2)	96.00	3.33	4.44	0.97	1.54
PFOW-MoE(with weak signal detection) (top-k = 3)	96.00	3.33	4.44	0.97	2.31

## Data Availability

The original contributions presented in this study are included in the article. Further inquiries can be directed to the corresponding author.
